# *Buteo* Nesting Ecology: Evaluating Nesting of Swainson’s Hawks in the Northern Great Plains

**DOI:** 10.1371/journal.pone.0137045

**Published:** 2015-09-01

**Authors:** Will M. Inselman, Shubham Datta, Jonathan A. Jenks, Kent C. Jensen, Troy W. Grovenburg

**Affiliations:** Department of Natural Resource Management, South Dakota State University, Brookings, South Dakota, United States of America; University of Regina, CANADA

## Abstract

Swainson’s hawks (*Buteo swainsoni*) are long-distance migratory raptors that nest primarily in isolated trees located in areas of high grassland density. In recent years, anthropogenic conversion of grassland habitat has raised concerns about the status of the breeding population in the northern Great Plains. In 2013, we initiated a study to investigate the influence of extrinsic factors influencing Swainson’s hawk nesting ecology in north-central South Dakota and south-central North Dakota. Using ground and aerial surveys, we located and monitored nesting Swainson’s hawk pairs: 73 in 2013 and 120 in 2014. We documented 98 successful breeding attempts that fledged 163 chicks; 1.52 and 1.72 fledglings per successful nest in 2013 and 2014, respectively. We used Program MARK to evaluate the influence of land cover on nest survival. The top model, *S*_Dist2Farm+%Hay_, indicated that nest survival (fledging at least one chick) decreased as nests were located farther from farm sites and as the percent of hay cover increased within 1200-m of the nest site (34.4%; 95% CI = 27.6%–42.3%). We used logistic regression analysis to evaluate the influence of landscape variables on nest-site selection; Swainson’s hawks selected for nest sites located closer to roads. We suggest that tree belts associated with farm sites, whether occupied or not, provide critical breeding sites for Swainson’s hawks. Additionally, poor breeding success may be related to the late migratory behavior of this species which requires them to occupy marginal habitat due to other raptors occupying the most suitable habitat prior to Swainson’s hawks arriving to the breeding grounds.

## Introduction

Swainson’s hawks (*Buteo swainsoni*) are long-distance migratory raptors that nest primarily in areas consisting of isolated tree stands in open grassland areas [1–3]. Due to the broad distribution of Swainson’s hawks across much of the central and western United States and Canada, numerous studies have been conducted documenting reproduction across their range [1, 2, 4–9]. Swainson’s hawks nest in high densities in the Prairie Pothole Region of the Great Plains [1, 10–12]. However, continued grassland loss has resulted in the Swainson’s hawk being listed as a Species of Concern by state and federal agencies [11–13].

In the northern Great Plains, extrinsic factors influencing nest survival of Swainson’s hawks have received little attention [1]. These factors related to habitat, predation, competition, and climate have the potential to positively [3, 14] or negatively [15] affect nest success rates. Changes in habitats surrounding nest sites could impact survival (e.g., displacing prey communities, increasing or changing predator populations, or increasing competition). Farming and ranching practices on remaining grasslands also are a potential concern; increased cattle production and infrequent haying could alter foraging habitats [16]. However, agriculturally rich habitats may increase productivity rates more than habitats lacking agriculture and potentially provide a stabilized prey base [17, 18–19]. Estimates of grassland lost from 2007–2013 were 1,202,000 ha in North Dakota and South Dakota [19−20]. Continued expansion of intensive agricultural practices raises concerns about potential impacts on nesting of grassland raptors (e.g., [9]).

Swainson’s hawks have been documented nesting in areas dominated by grasslands [1–2] as well as agriculturally dominated landscapes [1, 3, 21–22]; however, limited information exists concerning the influence of habitat variables on nest site selection in the northern Great Plains. Research conducted in intensively farmed areas has documented that Swainson’s hawks have increased productivity compared to Swainson’s hawks nesting in areas with lower intensities of agriculture [1, 14, 21–22]. The effects of specific crop types (e.g., row crop, small grain crop) on nest survival and nest site selection are currently unknown. Previous studies have focused on nest site characteristics and habitat around the nest on a micro- scale (e.g., [9]). Evaluating the effects of habitat on a larger scale (e.g., home range), could provide additional understanding of land cover effects on nest survival and nest site selection [5, 15].

The first objective of this study was to evaluate the influence of extrinsic (e.g., percent row crop, distance to farm) variables on nest survival of Swainson’s hawks in the northern Great Plains. Substantial conversion of grassland to row crops has occurred over the past 10 years [19–20]; therefore, we predicted that nest survival of Swainson’s hawks would be negatively affected by row crops whereas grassland nearer nest sites would positively influence nest survival. Our second objective was to evaluate the influence of habitat variables on nest site selection. We predicted the increase in row crop fields and the lack of trees on this landscape would lead Swainson’s hawks to select for areas with high percentages of grassland and trees while selecting against areas of row crop agriculture.

## Materials and Methods

### Study Area

**Fig 1:**
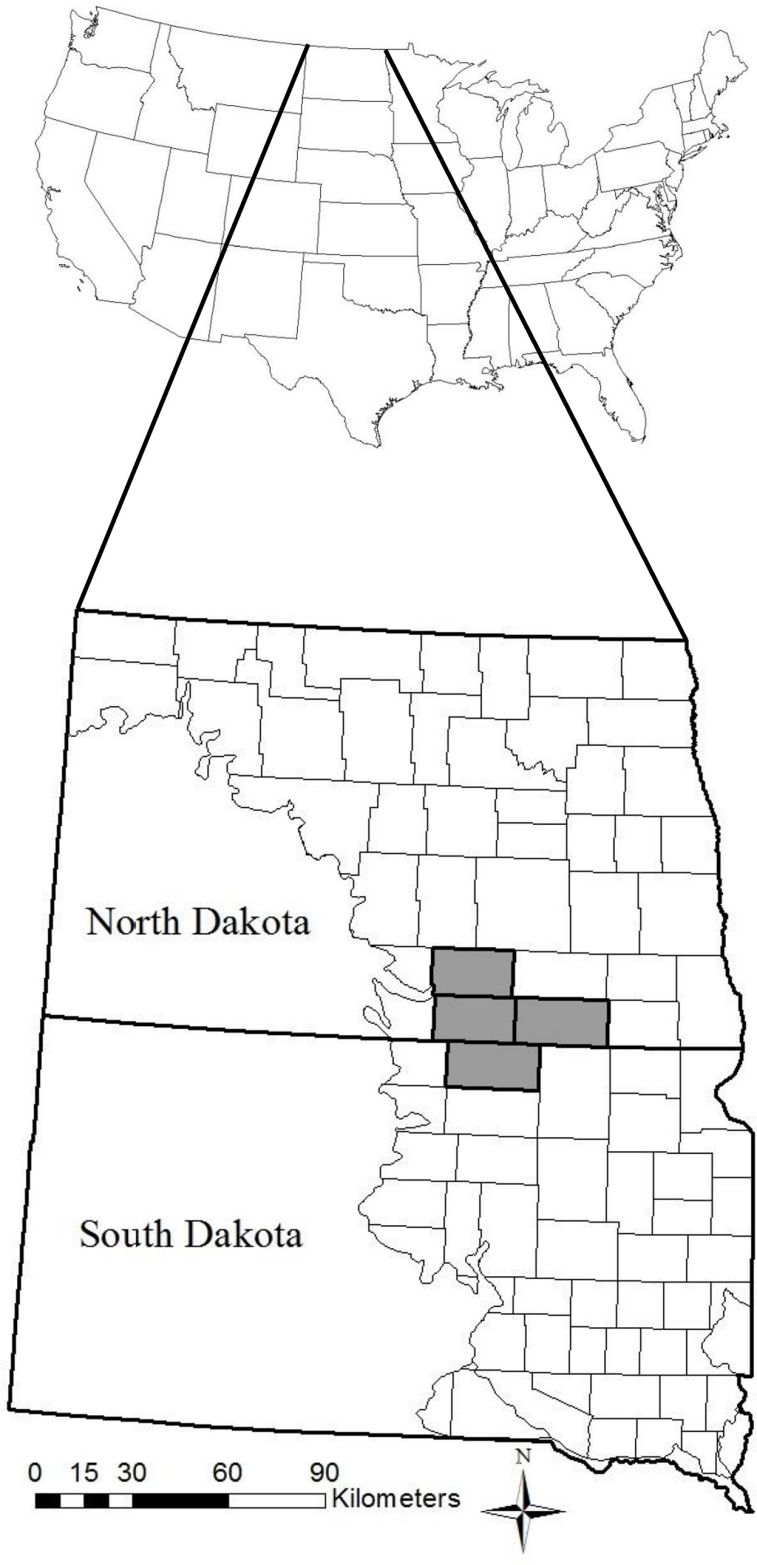
Swainson’s hawk nest ecology study area in south-central North Dakota and north-central South Dakota, USA, 2013–2014. Swainson’s hawk (*Buteo swainsoni*) study area (shaded) in Logan, McIntosh, and Dickey counties, North Dakota and McPherson County, South Dakota, USA, 2013–2014.

The 11,137 km^2^ study area consisted of four counties located in south-central North Dakota and north-central South Dakota (Fig 1). McPherson County, South Dakota and Dickey, McIntosh, and Logan counties, North Dakota, lie within the Northern and Northwestern Glaciated Plains level III ecoregion [23]. This moraine landscape contains numerous pothole wetlands scattered among the rolling terrain, which is typical of the Missouri Coteau region [10, 23]. Land use in the four counties consisted of cultivated land (62.5%), grassland (17.4%), and development (13.7%), with the remaining land consisting of forested cover (3.6%) and wetlands (2.8%; [24]). Average high and low temperatures for the months of April through July ranged from 11.6°C to 29.3°C and –0.5°C to 14.4°C, respectively [25]. Average annual precipitation was 45–53 cm and the majority of precipitation events occurred during May to September [25]. Dominant vegetation consisted of western wheatgrass (*Pascopyrum smithii*), green needlegrass (*Nassella viridula*), northern reedgrass (*Calamgrostis stricta*), prairie cordgrass (*Spartina pectinata*) big bluestem (*Andropogon gerardi*), western wheatgrass (*Pascopyrum smithii*), porcupine grass (*Stipa spartea*), and little bluestem (*Schizachyrium scoparium*; [23]). Tree species were primarily eastern cottonwood (*Populus deltoides*), American elm (*Ulmus americana*), box-elder (*Acer negundo*), and green ash (*Fraxinus pennsylvanica*; [10]).

### Nest Monitoring

We began searching for active nests on 1 May of each breeding season (2013 and 2014) targeting all tree sites (e.g., shelterbelts, farmsteads, riparian areas) in the study area. We attempted to locate all active Swainson’s hawk nest structures from roads before tree foliage obscured our ability to locate nests. If we located a breeding pair when tree growth obscured our view, we gained landowner permission and located nest sites by foot. We used vehicles to systematically drive all accessible roads in each county; roads that were not accessible by vehicle were traveled on foot. We used aerial surveys to cover remaining areas inaccessible by vehicle or foot. We considered nest sites active if there was evidence of nesting behavior (e.g., copulation, incubation; [1]). All active nest site locations were recorded using handheld Garmin GPSMAP 62 Global Positioning System (GPS; Garmin Ltd.) units and were then entered into ArcGIS 10.1 [26]. We monitored nest sites from roads (distance ≤600 m) using binoculars and spotting scopes at least once every two weeks throughout each breeding season (1 May–15 Aug). When the nestlings became visible in the nests, we entered nest structures using ladders or climbing equipment. At each nest we recorded the number of nestlings and each chick was then fitted with a numbered aluminum United States Fish and Wildlife Service lock-on band if they were ≥14 days of age. The species of the nest tree was identified, and we used clinometers and rangefinders to estimate nest height above the ground and the height of the nest tree.

Our nest monitoring protocol for this study followed the guidelines established by [27], all animal handling methods followed the guidelines approved by The Ornithological Council [28] and were approved by the Institutional Animal Care and Use Committee at South Dakota State University (Approval No. 13-002A). Data collection and those data collected on public land were authorized by South Dakota Game, Fish, and Parks, North Dakota Game and Fish, and United States Fish and Wildlife Service. Access to private lands was granted by individual landowners for data collection. No endangered or threatened species were involved in this study.

### Statistical Analysis

#### Habitat measurements

We used the Cropland Data Layer (CDL; [24]) to evaluate land cover at nest sites. We reclassified the CDL layers from 2013 and 2014 for each state to represent the land cover variables we assessed as biologically significant from published literature [3]; row crop, grain crop, alfalfa/hay, grassland, water, trees, and farm sites. We generated random points using the Random Point Generator tool in ArcGIS 10.1 to simulate random nest sites for logistic regression analysis. If a generated random point was not located at a visible tree, it was repositioned to the nearest available tree to simulate a nest site. We clipped reclassified CDL layers to 1200-m buffers around each random and nest site using Geospatial Modeling Environment [29] and calculated land cover percentages for extrinsic variables using ArcGIS 10.1. We selected the 1200-m (4.5 km^2^) buffer based on the median range of breeding territory size for Swainson’s hawks in this region (6.4 km^2^–0.01km^2^; [30]). For nest survival, we also assessed distance to landscape features (meters); distance to farms, distance to wetlands, and distance to roads using ArcGIS 10.1. We used the Focal Statistics tool in the Spatial Analyst package to calculate the number of inter- and intraspecific raptor nests within the 1200-m buffers. We identified two other raptor species, red-tailed hawks (*Buteo jamaicensis*) and ferruginous hawks (*Buteo regalis*), as interspecific competitors to Swainson’s hawks. These nest were located and monitored similarly to that of the Swainson’s hawk nests. All statistical tests were conducted using program R [31] with an experiment-wide error rate of 0.05.

#### Nest survival analysis

We selected a suite of 12 predictor variables from field observations consisting of land cover, distance to landscape features, and number of nearest raptor nests as potential factors effecting nest survival (Table 1). We used Pearson’s correlation for evidence of multicollinerity and excluded covariates from the same model if *r* ≥ |0.7|. We considered nests successful if they fledged ≥1 young and used nest survival models in Program MARK [32] with the logit-link function to evaluate the effect of predictor variables on nest survival throughout the nesting season. We created 17 models (Table 2) from field observations that we believed were biologically significant and used Akaike’s Information Criterion (AIC_*c*_) corrected for small sample size to select models that best described the data [33]. We considered models as competing models if they differed by ≤2 ∆AIC_*c*_ [33] from the top model and used Akaike weights (*w*_*i*_) as an indication of support for each model. We evaluated whether competing models contained covariates where β-estimates did not have 95% confidence intervals that encompassed zero [34–35]. There is currently no goodness-of-fit test for nest survival; therefore, we investigated model robustness by artificially inflating ĉ (i.e., a model term representing over dispersion) from 1.0 to 3.0 (i.e., no dispersion to extreme dispersion) to simulate various levels of dispersion reflected in Quasi-AICc (QAICc; [35–36]).

**Table 1:** Final variables measured within 1200-m buffers of nest sites used to model the influence of intrinsic and extrinsic factors on Swainson’s hawk nest survival and nest site selection in the northern Great Plains, USA, 2013–2014.

Variable Name	Definition
Row Crop	Total corn and soybean cover (%)
Grain Crop	Total wheat and oat crop cover (%)
Hay	Total alfalfa/grass hay cover (%)
Grass	Total disturbed and undisturbed grassland (%)
Water	Total wetland cover (%)
Trees	Total tree cover (%)
Farm Sites	Total area occupied by farm house and outbuildings including associated trees (%)
Distance to farm	Distance to nearest farm site (m)
Distance to road	Distance to nearest road (m)
Distance to wetland	Distance to nearest wetland (m)
Number of nearest raptor nests	Number of raptor nests within 4.5 km^2^ of nest site

**Table 2:** Nest survival models of Swainson’s hawks during the 2013–2014 breeding season in South Dakota and North Dakota, USA.

Model	AIC_*c*_ ^a^	∆AIC_*c*_ ^b^	*w*_*i*_ ^c^	*K* ^d^	Deviance
*S*_Dist2Farm+%Hay_	569.09	0.00	0.59	3	563.09
*S*_%GrainCrop+%Hay+%Farm Sites+Dist2Farm+#NearestRaptorNests_	572.33	3.24	0.12	6	560.32
*S*_%Farm Sites+Dist2Farm_	573.01	3.92	0.08	3	567.01
*S*_Dist2Farm_	573.03	3.94	0.08	2	569.03
*S*_#NearestRaptorNests+Dist2Farm_	573.73	4.64	0.06	3	567.72
*S*_%Farm Sites_	576.44	7.35	0.02	2	572.44
*S*_Dist2Road_	577.02	7.93	0.01	2	573.02
*S*_Constant_	577.43	8.34	0.01	1	575.43
*S*_Saturated Model_ ^e^	577.75	8.66	0.01	13	552.71
*S*_%Hay+%Grass+%Trees_	577.78	8.68	0.01	4	569.77
*S*_#NearestRaptorNests_	578.15	9.06	0.01	2	574.15
*S*_Dist2Water_	579.41	10.32	0.00	2	575.41
*S*_%Water_	579.43	10.34	0.00	2	575.43
*S*_%RowCrop+%GrainCrop+% Farm Sites_	579.76	10.67	0.00	4	571.76
*S*_%RowCrop+%GrainCrop_	580.20	11.11	0.00	3	574.20

^a^ Akaike’s Information Criterion corrected for small sample size (Burnham and Anderson 2002).

^b^ Difference in AICc relative to min. AIC.

^c^ Akaike wt (Burnham and Anderson 2002).

^d^ Number of parameters.

^e^ Saturated Model = Contains all variables measured during the study.

#### Nest site selection

We used logistic regression and Akaike’s Information Criterion (AIC) to determine the effects of intrinsic and extrinsic variables on nest site selection. We generated 190 random nest sites to use as pseudo-absent points. We created 11 *a priori* models from published literature (Table 3; [1, 3]) to estimate the influence of our selected predictor variables (Table 1). We considered models as competing models if they differed by ≤2 ∆AIC [34] from the top model and used Akaike weights (*w*_*i*_) as an indication of support for each model. Predictive capacities of significant models were tested using receiver operating characteristics (ROC) values. We followed guidelines stated by [37] and considered acceptable discrimination for ROC values between 0.7 and 0.8 and excellent discrimination between 0.8 and 1. We used logistic odds-ratios to evaluate the effect of variables in the optimal model on nest site selection.

**Table 3:** Akaike’s Information Criterion (AIC) model selection of logistic regression models for nest site selection of Swainson’s hawks in South Dakota and North Dakota, USA, 2013–2014.

Model Covariates	*K*	AIC	∆AIC	*w*_*i*_	ROC^a^
Trees + Dist2Road	3	318.72	0.00	0.57	0.93
Water + Trees + Dist2Road	4	319.01	0.59	0.43	0.91
RowCrop + Hay + Dist2Farm	4	420.38	101.96	4.14E-23	0.73
RowCrop + Trees + Farm Sites + Dist2Wetland	5	422.84	104.81	9.96E-24	0.74
Hay + Dist2Farm	3	424.58	105.87	5.87E-24	0.77
Water + Dist2Farm	3	427.00	108.28	1.75E-24	0.82
RowCrop + GrainCrop + Hay + Dist2Wetland + Trees + Farm Sites	7	425.37	108.38	1.67E-24	0.64
Trees + Water + Grass + Year	5	428.93	110.89	4.75E-25	0.77
RowCrop + Water + Trees	4	437.13	118.71	9.52E-27	0.68
Grass + Hay + Trees	4	438.89	120.47	3.96E-27	0.70
Trees + Dist2Wetland	3	440.28	121.56	2.29E-27	0.79

^a^ ROC = receiver operating characteristic curve. Values between 0.7–0.8 considered acceptable discrimination and between 0.8–1 were considered excellent discrimination (Hosmer and Lemeshow 2000).

## Results

We located and monitored Swainson’s hawk nests in south-central North Dakota and north-central South Dakota: 73 in 2013 and 120 in 2014. Breeding adults were observed arriving on the study area on 28 April 2013 and 26 April 2014. In 2013 we documented 29 successful breeding attempts that produced 44 fledglings. In 2014, 69 successful breeding attempts produced 119 fledglings. Swainson’s hawks fledged 1.52 and 1.72 fledglings per successful nest in 2013 and 2014, respectively.

Nest survival analysis indicated that model *S*_Dist2Farm+%Hay_ was the top-ranked model (*w*_*i*_ = 0.59), and indicated that nest success increased when nests were closer to farmsteads and in areas with lower percent hay land. (Table 2). The 95% confidence intervals of the β estimates for Dist2Farm (−0.0003, 95% CI = −0.0006 to −0.0001) and %Hay (−0.03, 95% CI = −0.06 to −0.007) did not encompass zero; using this model nest survival was 34.4% (95% CI = 27.6%–42.3%). When adjusting ĉ from 1.0 to 3.0 to test for over dispersion, interpretation of our top model *S*_Dist2Farm+%Hay_ did not change and it remained the top-ranked model when ĉ = 2.0 (moderate dispersion; QAIC_c_ wt = 0.49) and through ĉ = 3.0 (extreme dispersion; QAIC_c_ wt = 0.33).

At 193 nest sites, American elm (47%) was the most common tree species used followed by green ash (22%), eastern cottonwood (17%), and box elder (6%). Eastern red-cedar (*Juniperus virginiana*), peachleaf willow (*Salix amygdaloides*), Russian olive (*Elaegnus angustifolia*), and chokecherry (*Prunus virginiana*) accounted for the remaining 9% of nest trees. Average tree height used for nesting was 10.9 m (*n* = 132, SE = 0.56) and nest height averaged 9.0 m (*n* = 113, SE = 0.54). The highest recorded nest was 23.4 m (eastern cottonwood) and the lowest recorded nest height was 1.7 m (peachleaf willow).

Model [Trees + Dist2Road] was the top-ranked model (*w*_*i*_ = 0.57) for predicting nest site selection of Swainson’s hawks; predictive capability of the model was excellent (ROC = 0.91; Table 3). Logistic odds-ratio estimates from the top-ranked model indicated the odds of nest site selection were 0.99 (95% CI = 0.98–0.99) times less likely for every meter increase from the nearest road. Although the percentage of trees was included in the top-ranked model, the logistic odds ratio (0.69, 95% CI = 0.45–1.04) did not differ from one indicating no effect. A second competing model was observed from the model results; [Water + Trees + Dist2Road] (*w*_*i*_ = 0.42). Similar to the top model, logistic odds ratios indicated that Dist2Road (0.99, 95% CI = 0.98−0.99) was the only significant variable influencing nest site selection as logistic odds ratios for variables water (0.97, 95% CI = 0.94−1.01) and trees (0.74, 95% CI = 0.47−1.13) did not differ from one. Thus this model was not considered as a competing model for additional justification of nest site selection (Table 3).

## Discussion

Our results suggest that reproductive success of this breeding population of Swainson’s hawks is relatively low. The nest survival estimate during our study was lower than previously documented (81%; [2], 48%; [4], 44–58%; [9]), though available habitat varied between our study and similar reproductive success studies. Our study contained more land dedicated to row crop production than studies conducted in Arizona [9], New Mexico [2], or Colorado [4]. Nest survival results indicate that this population is currently declining in the northern Great Plains which is contrary to current research that indicates increasing or stable Swainson’s hawk populations in other parts of North America (e.g., [9]).

Our analysis indicated that distance to the nearest farm site and the percent of hay land had the greatest influence on nest survival. Nests that were located closer to farm sites had an increased probability of survival. Swainson’s hawks were frequently observed nesting near or within farm sites in our study similar to Swainson’s hawks in central North Dakota [1]. Even though the number of farms have decreased 18% in South Dakota and North Dakota from 1980–2009 [38], existing farm sites seem to provide breeding habitat for Swainson’s hawks by providing mature trees for nesting, which was previously documented by Gilmer and Stewart [1]. Farm sites also may provide suitable foraging habitats (e.g., frequently mowed grass increasing prey vulnerability); thus, farm sites may be a potential limiting factor for Swainson’s hawks in this region. Farm sites may provide a niche that is unoccupied by other predators (i.e. red-tailed hawks); predators/competitors may already occupy higher quality habitats (e.g., [21]) and may avoid these sites due to frequent human disturbance (e.g., daily farming operations). Swainson’s hawks arrive on the breeding grounds later than other competitors (i.e. red-tailed hawk and ferruginous hawk; [39]), which may require them to establish breeding territories in less than suitable habitat potentially contributing to their low nest survival rates.

Hay cover around nest sites negatively influenced nest survival during our study. Contrary to our findings, Swainson’s hawks have been documented selecting for hay fields around nest sites [9, 40]. Our study area contained other habitats that were available for foraging (e.g., grassland, pasture, farm sites) compared to Swainson’s hawks in California that selected for alfalfa and fallow fields in a tree-crop dominated landscape [40]. Grasslands and other non-row crop fields around nest sites may provide access to prey as the summer progresses and vegetation height obstructs Swainson’s hawk foraging abilities [39, 41]. Prey accessibility has been hypothesized to drive Swainson’s hawk foraging rather than prey densities in a particular habitat which is driven by vegetation height [39, 41]. We found that Swainson’s hawks in our study nested in areas of relatively low hay cover. However, we observed Swainson’s hawks switching to foraging primarily in hay fields when vegetation height in other habitats made them inaccessible (e.g., row crops, grain crops) for hunting, particularly during the brood rearing period (25 Jun–15 Aug). However, additional research on prey accessibility is needed to understand the magnitude of this effect on Swainson’s hawk nest survival in our study area.

Swainson’s hawks in our study established nest sites that were dominated by grassland (Table 4) similar to Swainson’s hawks in central North Dakota [1]. Significant land use change has occurred over the last 30 years which has altered the amount of available high quality habitat for these birds [14–15]. Gilmer and Stewart [1] estimated that the amount of cultivated crops within their study area accounted for 36% of available land cover, which is almost half of the current amount of available cultivated crop land currently in production in our study area (Table 4; 62.5%). However, Swainson’s hawks in our study still nested in grassland dominated areas. Conversely, research conducted in southeastern Alberta indicated productivity of Swainson’s hawks was actually higher in agriculturally rich areas [16, 19]. However, our results indicate there may not be a benefit from nesting in agriculturally dominated areas in our region; land cover around nest sites only contained one-third cultivated crops in a landscape that is comprised of two-thirds cultivated land.

**Table 4:** Mean and standard error (SE) for land cover and distance to landscape features for Swainson’s hawk nests in north-central South Dakota and south-central North Dakota, USA, 2013–2014.

	All SWHA Nests	
	(*n* = 193)	
Variable Name	$\bar{X}$	SE
Row Crop (%)	25.60	1.43
Grain Crop (%)	7.24	0.62
Hay (%)	9.15	0.52
Grass (%)	47.24	1.40
Water (%)	6.37	0.53
Trees (%)	0.33	0.04
Farm Sites (%)	3.95	0.11
Distance to Wetland (m)	353.47	25.30
Distance to Road (m)	132.04	8.58
Distance to Farm (m)	812.72	55.31
Number of Nearest Raptor Nests	1.63	0.17

We documented poor reproductive success during our study, which could have been attributed to intrinsic factors not measured in our analysis. Gilmer and Stewart [1] attributed a substantial amount of nest failures to hail and wind, however, we suspect that disease (i.e., West Nile virus; WNv) is potentially contributing to the low nest success during our study. Disease is an intrinsic factor of interest because of its lethality to nestling raptors [42−43]. Although no population level effects were documented by Stout et al. [42], low percentage of WNv antibodies in nestlings, may have short-term impacts on nest survival. Concurrent research conducted in this study area documented cases of WNv in ferruginous hawk fledglings [44]. Additionally, nest cameras from a concurrent study [31] displayed Swainson’s hawk chicks exhibiting similar WNv symptoms (e.g., lethargy, head-bobbing, lack of appetite) experienced by the ferruginous hawk chicks before their subsequent death. However, due to rapid decomposition, we were not able retrieve the carcasses to confirm cause-specific mortality. Disease still remains a serious conservation concern for this species as immediate affects could be detrimental to this declining population.

A majority of the Swainson’s hawk nests in our study where constructed in American elm trees. These findings are different than previous research in south-central North Dakota that documented American elms as accounting for less than 1% of the nest trees used by Swainson’s hawks [1]. Eastern cottonwood trees, which made up 45% of nest trees used in 1977–79 [1], only accounted for 17% of nest sites in our study. Shelterbelts in this region consisted primarily of American elm and green ash; nest tree selection reflected this availability, whereas eastern cottonwoods were located primarily in isolated patches around or near wetlands. Wetlands have declined by 7.4% the last 25–32 years across the Dakota Prairie Pothole Region (eastern North Dakota and South Dakota; [45]) due to agricultural expansion. This factor may have contributed to a shift in nest tree species used for nest sites since 1984 [1].

Nest site selection results indicate that Swainson’s hawks in our study selected nest sites near roads with great propensity, similar to Swainson’s hawks in central North Dakota [1]. Access to grassy road right-of-ways, which may provide favorable small mammal habitat, and roads, which increase prey vulnerability, create foraging habitats similar to farm sites that make up for the lack of available foraging habitat. While, nest survival analysis did not indicate a negative effect of distance to road on nest survival, these areas may be occupied by Swainson’s hawks because of the lack of suitable habitat elsewhere and may expose them to a greater risk of mortality (i.e., collisions with vehicles and human persecution). Roads and road right-of-way habitats may function as sink habitats for Swainson’s hawks.

## Conclusion

Our study provides updated information on nesting ecology of Swainson’s hawks in the northern Great Plains; a landscape that has undergone significant land use changes in the last decade. Distance to farm and percent hay cover explained some of the variation in our low estimates of nest survival. However, there may be underlying biological or environmental factors affecting overall nest survival (i.e., disease). Swainson’s hawks selected for nest sites that were located near roads, which may provide them suitable habitat. However, this road habitat may be acting as a sink contributing to low nest survival rates. This habitat potentially simulates historic grassland territories previously occupied by Swainson’s hawks, before they were excluded from high-quality habitats due to high raptor nesting densities and row crop agriculture expansion. Likewise, the late arriving migratory behavior of this species may be requiring them to make use of any available habitat whether suitable or not. We suggest that farmsteads, whether occupied or not, may provide critical breeding sites as suitable habitats are already occupied by other raptors. Migration strategies of Swainson’s hawks (i.e., late arrival to nesting grounds) coupled with high densities of nesting interspecific raptors may be contributing to the decline of this species in northern Great Plains.
